# Role of Biomarkers in the Clinical Management of Glioblastomas: What are the Barriers and How Can We Overcome Them?

**DOI:** 10.3389/fneur.2012.00188

**Published:** 2013-01-18

**Authors:** Kerrie L. McDonald, Grace Aw, Paul Kleihues

**Affiliations:** ^1^Lowy Cancer Research Centre, Prince of Wales Clinical School, University of New South WalesKensington, Australia

**Keywords:** biomarkers, treatment response, glioblastoma, MGMT, tumor heterogeneity

## Abstract

Thousands of articles describing biomarkers predictive of treatment and prognostic of survival in cancer have been published, yet only a handful of biomarkers are currently used routinely in the clinic. Biomarkers need to be analytically standardized, validated, and clinically useful. This review will address the challenges and ways in which we can improve our discovery and translation of prospective biomarkers from the lab into validated diagnostic tests with a specific focus on patients diagnosed with glioblastoma and *MGMT* promoter methylation status. There has been long-held enthusiasm to use *MGMT* promoter methylation as a predictive biomarker for patients treated with the alkylating agent, temozolomide; however in the majority of centers around the world, this has not yet transpired.

## Introduction

The idea that we should be treating cancer patients on an individual basis has become a major objective in clinical oncology (Jain, 2002). In the past 10 years, more than 200,000 articles describing biomarkers that predict treatment outcome have been published. However, few biomarkers are currently used routinely in the clinic. One very clear lesson learned is that anecdotal, underpowered biomarker studies have little clinical value and tend to significantly slow down progress. Introduction into the clinic before appropriate validation is costly and can result in the under treatment or over treatment of patients (Dammann and Weber, 2012). The translation of prospective biomarkers from the lab into validated diagnostic tests is a major challenge. This review will focus upon the discovery and development of biomarkers in glioblastoma (GBM).

## Definition of a Biomarker

The critical hallmark of a biomarker is to be highly sensitive and specific in providing information relevant for diagnosis, prognosis, or therapy. There is no strict rule when it comes to what constitutes a biomarker. A marker can consist of alterations of the genome, epigenome, transcriptome, or proteome, aberrant microRNA as well as imaging changes observed on a MRI or PET scan. The most sought after biomarkers are the ones that can identify which patients are at high risk of tumor relapse, and cytotoxicity in response to specific chemotherapeutic agents. In addition, biomarkers that can be easily ascertained by simple and inexpensive methodologies such as immunohistochemistry (IHC) are also in demand. The use of biomarkers to identify patients who do not respond to treatment early could confer enormous benefits for patients diagnosed with GBM, especially considering their usually short survival time. A few biomarkers have shown excellent utility in survival prognostication but not necessarily at the level of influencing an oncologist’s decision to administer a specific drug or alter the treatment schedule. A summary of the key biomarkers used for the diagnosis, prognosis and predicting treatment response in GBM is provided in Table 1.

**Table 1:** **Biomarker summary**.

Biomarker	Standardized Test?	Diagnostic Use?	Prognostic Use?	Predictive Use?	Druggable Target?
MGMT promoter methylation	No	No	Yes	Yes	No
	MSP, pyrosequencing, RT-PCR		Anaplastic glioma	Elderly GBM	

IDH1/2 mutation	Yes	Yes	Yes	No	No
	IHC (IDH1-R132H). Sequencing	Can aid in the differential diagnosis of grade II/III glioma from pilocytic astrocytoma, ependymomas and reactive gliosis Can detect the infiltration of tumor cells in normal tissue	Prognostically favourable Indicative of secondary GBM		Mutant IDH1 is considered to be a potentialtherapeutic target; however no treatments are available as yet

TP53mutation	No	Yes	No	No	No
	IHC is commonly used however this is not representative of a mutation	Genetic hallmark in 60-70% of low-grade diffuseastrocytomas, anaplastic astrocytomas and secondary GBMs	Some studies have found a shorter time interval to progression in patients with TP53 mutant low-grade astrocytoma

EGFR amplification	Yes	Limited	No	No	Yes
	IHC does not differentiate between EGFR amplification and overexpression	EGFR amplification indicates primary GBM			Cetuximab Erlotinib Gefitinib Gefitinib EGFRvIII: rhindopepimut

VEGF	No	No	No	No	Yes
	IHC, but not used clinically				bevacizumab

## Diagnostic Biomarkers

### Biomarkers to distinguish primary GBM from secondary GBM

Primary GBM develops very fast *de novo*, without clinical or histopathological evidence of a less malignant precursor lesion (Scherer, 1940). Secondary GBM develops from low-grade diffuse astrocytoma (LGA, WHO grade II) or anaplastic astrocytoma (AA, WHO grade III). The pace of progression to a secondary GBM varies considerably. The mean interval from LGA to secondary GBM is approximately 5 years; from AA to secondary GBM is approximately 1.5–2 years.

From population-based clinical observations it has been estimated that approximately 5% of all GBMs are secondary. However, progression to secondary GBM may occur fast or asymptomatic and thus escape clinical detection. They constitute a distinct tumor entity with a significant difference in age distribution. Primary GBMs affect the elderly, with a mean age at diagnosis of approximately 60 years. Secondary GBM patients are approximately 15 years younger. They have a preferential location in the frontal lobe, a lower Male/Female ratio and a significantly better clinical outcome. Histopathologically, these two subtypes of GBM cannot reliably be distinguished, however their genetic profile differs significantly (Watanabe et al., 1996; Ohgaki et al., 2004).

The detection of mutations in the Krebs cycle enzyme *isocitrate dehydrogenase (IDH)* has become a reliable molecular test for identifying secondary GBM. Using high-throughput next generation sequencing, recurrent mutations in the active site of IDH1 have been identified in approximately 12% of all GBM and were typically observed in younger patients with secondary GBM (Parsons et al., 2008). Since the IHC test for *IDH* mutation detection was introduced to the clinic, secondary GBMs have been shown to constitute approximately 10% of all GBMs (previously estimated at 5% on the basis of a known precursor lesion and age; Nobusawa et al., 2009). Point mutations at the arginine 132 (IDH1) and at arginine 140 or arginine 172 (IDH2) in the active site of IDH enzyme alter catalytic activity of the enzyme and results in increased production of 2-hydroxyglutarate (2-HG) which is potentially associated with increased cancer risk and glioma progression (Dang et al., 2009). A recent study showed that 2-HG also had a potential to competitively inhibit α-ketoglutarate-dependent enzymes including histone demethylases and 5-methylcytosine hydroxylases which in turn leads to genome-wide histone modifications and DNA methylation changes (Xu et al., 2011).

Additionally, mutations in the tumor suppressor *TP53* are observed in 70% of secondary GBM (Ohgaki and Kleihues, 2007). *TP53* negatively regulates the proliferation of cells and plays an important role in the transcriptional activation or repression of several genes, in particular *p21*, an inhibitor of cyclin-dependent kinases [CDKs]). Through this pathway, *TP53* mediates cell cycle control at G1/S and G2/M checkpoints (Agarwal et al., 1995). Many laboratories use IHC to detect TP53 expression, but in CNS tumors, immunoreactivity is not representative of the gene mutation status.

## Prognostic Biomarkers

### Classical phenotypical traits

By definition, a prognostic marker has the capacity to estimate survival outcome, independent of whether or not the patient receives adjuvant radiation therapy (RT) and/or chemotherapy. Classical phenotypical traits that are prognostic for survival include age, performance status, tumor location, and histological grade. Older age has been consistently associated with poor prognosis. In a historical population-based study, there was a linear decrease of overall survival with increasing age. Patients aged 70–79 years had a median survival of 2.9 months, those above 80 years only 1.9 months (Ohgaki et al., 2004). Similarly, a high preoperative performance status has a favorable prognostic impact, irrespective of subsequent adjuvant therapy (Lacroix et al., 2001; Whittle et al., 2002; Buckner, 2003). Size and location are also highly prognostic, with the worst outcome for GBMs being tumors that have spread extensively, e.g., across the corpus callosum to the contralateral hemisphere (Buckner, 2003).

### Using genomic changes in GBM as a diagnostic tool to select patients for targeted therapy

GBM is highly heterogeneous. The 2007 World Health Organization (WHO) classification of tumors lists giant cell GBM and gliosarcoma as histological variants of GBM. These rare subtypes reflect the marked genomic instability of the tumors. However the variable survival times of patients suggests that there are multiple subtypes of GBM. The genetic profiling of large tumor cohorts with comprehensive clinical and survival data has promoted the discovery of novel molecular biomarkers associated with survival, in addition to traditional clinical and morphological features (Nutt et al., 2003; Rich et al., 2005; Colman et al., 2010; Verhaak et al., 2010). In GBM, comprehensive gene sequencing performed by The Cancer Genome Atlas (TCGA) has identified an average of 47 mutations per tumor, although far fewer were candidates to be driver mutations (Parsons et al., 2008). Driver mutations were most frequent in the TP53, RB1, and PI3K/PTEN pathways. Mutations in these pathways were generally mutually exclusive, suggesting that they are key to tumorigenesis, and functionally equivalent (Parsons et al., 2008).

At least two distinct cluster profiles have been identified: proneural and mesenchymal-angiogenic signatures, which differ in survival and response to treatment (Colman et al., 2010; Verhaak et al., 2010). Verhaak et al. (2010) performed consensus clustering on the results of TCGA gene expression arrays from 200 samples of GBM, proposing classification into not two, but four subtypes: proneural, neural, classical, and mesenchymal. Younger age and longer survival were common features of patients with proneural subtypes. Patients benefiting from concurrent chemotherapy were associated with the classical GBM subtype. The mesenchymal GBM subgroup was connected with poor survivorship. Similar frequently mutated genes were identified to previous work, including *TP53, PTEN, NF1, EGFR, IDH1, PIK3R1, RB1, ERBB2, EGFRvIII, PIK3CA*, and *PDGFRA*. They identified clinical correlations, for example with younger age and proneural subtype, as well as survival differences by treatment, such as benefits from combined chemoradiotherapy in the classical subtype which were not seen in the proneural subtype. Secondary GBMs with *IDH1/2* mutations have almost always a proneural signature.

Unfortunately no single classification is yet regarded as definitive, and our use of technology and understanding of what is clinically translatable continues to evolve (Vitucci et al., 2011). A comprehensive study of microarray data from four different independent data sets by Colman et al. (2010) identified a 38-gene signature associated with survival (31 genes associated with poor survival outcome). A smaller 9-gene signature based upon the 38 genes was tested on FFPE tissue from patients enrolled in the RTOG 0825 Phase III trial of newly diagnosed GBM.

There is a lack of confidence from biologists and diagnostic laboratories to use the 9-gene signature and/or to group GBM according to subtypes because the literature keeps changing. In a more recent study by Shen et al. (2012), three distinct GBM subtypes were identified using integrative subtype analysis of TCGA GBM dataset. There were overlaps between the Verhaak and Coleman studies but there were also striking differences. However, there is consensus in the neuro-oncology community to subtype GBM, even if it is into the broader groups of proneural and mesenchymal or suptype 1 and 3. Clinical trial efficacy with preselected patient populations will improve. It is anticipated that the *IDH1/2* mutational status will prove to be most reliable and significant.

## Genotype-Directed Therapy (Druggable Biomarkers)

### Epidermal growth factor receptor

Tumors belonging to the subtype 2 described by Shen and colleagues or the “classical” subtype coined by Verhaak and colleagues are associated with *Epidermal growth factor receptor (EGFR)* copy number amplification. This is the most common EGFR alteration found in 30–50% of GBM patients, leading to increased EGFR expression, activity of the EGF pathway, cell growth, and proliferation (Shinojima et al., 2003). EGFR amplified tumors can also harbor mutations, with the most common being an in-frame deletion of exons 2–7 (known as *EGFR vIII*; Pelloski et al., 2007). This deletion is present in 50–60% of GBM patients with EGFR amplification (Del Vecchio et al., 2012). EGFR overexpression detected by IHC is not representative of EGFR amplification, yet this test is commonly used.

Because of the frequency of EGFR aberrations, it is not surprising that there have been concerted efforts to therapeutically target EGFR. Inhibitors have been approved for use in non-small cell lung cancer (NSCL) and have gone to clinical trial in GBM; however the results have been very disappointing. Many patients with GBM either do not respond to these inhibitors from the outset and those patients that do respond, end up developing resistance. Cetuximab (Erbitux) has been shown to act synergistically in combination with cytostatic drugs and radiotherapy. More recently it has been shown that GBMs carrying a novel exon 27 deletion mutation (EGFR-CTD) respond better to cetuximab (Cho et al., 2011). Small molecule tyrosine kinase inhibitors (TKIs) including erlotinib (Tarceva^®^) and gefitinib (Iressa^®^) have also been investigated in GBM trials with disappointing outcomes. Recently these poor results have been attributed to the different conformational requirements of mutant EGFR in NSCL and GBM (Vivanco et al., 2012). EGFR inhibitors that target the active kinase conformation such as erlotinib are ineffective as a result of mutations or deletions in the extracellular domain resulting in EGFR addiction (Vivanco et al., 2012). NSCL susceptible to EGFR treatment tend to harbor mutations in the EGFR kinase domain.

The PTEN tumor suppressor gene also plays a key role in resistance to EGFR inhibitors. Loss or mutation of this gene occurs in ∼40% of GBM. Functional PTEN (retention of activity) has been linked to favorable response to erlotinib (Mellinghoff et al., 2005) however this could not be confirmed clinically (van den Bent et al., 2009). Additionally, phosphorylation of the conserved residue of Y240 in GBM has been shown to be associated with resistance to EGFR therapy (Fenton et al., 2012).

### Vascular endothelial growth factor

Vascular Endothelial Growth Factor (VEGF) is highly expressed in GBM and is a critical component of the pathophysiology (Folkman, 1995; Jain et al., 2007) and numerous experimental studies have supported a major role for VEGF in tumor angiogenesis (Dowlati and Fu, 2008)^.^ Blocking VEGF leads to impeded vessel growth and endothelial cell proliferation, vasoconstriction and vessel “normalization” which results in redistribution of blood flow in tumor tissue and improves delivery of anti-cancer agents to individual cells (McVicker and Tabatabai, 2006; Weiner et al., 2006). Bevacizumab targets VEGF-2, while the drugs cediranib (Wachsberger et al., 2011) and sunitinib (D’Amico et al., 2012) target also VEGFR-1, -2, and -3. The efficacy of cediranib was trialed in a Phase III study of recurrent GBM patients as a monotherapy and in combination with lomustine versus lomustine alone (REGAL study). Although there was evidence of activity, cediranib treatment did not improve survival (Ahluwalia, 2011).

The use of bevacizumab as a second line treatment for GBM was fast-tracked in the USA 3 years ago (Cohen et al., 2009; Cloughesy, 2010). FDA approval was based on positive results from two prospective phase II trials on patients with relapsed tumors (Vredenburgh et al., 2007; Friedman et al., 2009). Although close to half of patients treated with bevacizumab show a dramatic radiological response rate and an increased median progression free survival (PFS) compared to historic controls, *no impact on overall survival* has been reported (Friedman et al., 2009; Chamberlain, 2011).

### Summary of the current state of genotype-directed therapy

There is no doubt that genotype-directed medicine holds great promise for the treatment of tumors. However, we recognize that a multi-facetted, combination therapy approach will be necessary. In addition, what works in one cancer such as the EGFR inhibitors in NSCLC may not be effective in another solid cancer harboring the same mutation as a result of conformational changes. Additionally, there strong cross talk between the signaling pathways significantly adds to the complexities of genotype-targeted treatment of a tumor. Subtyping of GBM prior to clinical trial is essential. Clinical and research laboratories need to agree on a minimal molecular testing platform, whether it be the gene signatures or the testing of individual molecular markers and agreement on the methodologies must come into play.

### Biomarkers with treatment predictive value

Much more difficult to identify are biomarkers with predictive power in the context of a specific therapy. Predictive biomarkers are markers that can be used to identify groups of patients who are most likely to respond to a given treatment. The key difference between a prognostic and a predictive biomarker is that a predictive biomarker should instigate a *change* in the treatment provided to the patient.

### O^6^-methylguanine-DNA methyltransferase: Is methylation of MGMT predictive of response to temozolomide?

Detection of methylation within the promoter region of *O^6^-methylguanine-DNA methyltransferase* (*MGMT*) gene has clearly provided the most meaningful clinical correlation and is generally accepted as a predictive and prognostic biomarker in temozolomide treated GBM, even in elderly patients (Stupp et al., 2005a, 2009; Reifenberger et al., 2012; Wick et al., 2012). The *MGMT* gene is located on chromosome 10q26.1 and encodes a DNA repair protein that restores mutagenic O^6^-alkylguanine to normal guanine within genomic DNA. Alkylating drugs such as temozolomide are used in chemotherapy for the targeted cell death of rapidly replicating neoplastic cells and MGMT expression is a key factor in conferring resistance to these agents. Loss of MGMT protein expression is frequently associated with transcriptional silencing of the *MGMT* gene by methylation of its CpG island promoter in various neoplasia, (Esteller et al., 1999) as exemplified by 35–55% of gliomas (Silber et al., 1999; Esteller et al., 2001; Nakamura et al., 2001; Kamiryo et al., 2004; Brell et al., 2005; Hegi et al., 2005).

There has been considerable and long-held enthusiasm to use MGMT as a predictive biomarker for glioma patients, with the longer term hope for its use as a marker to assign therapy to individual patients. Three criteria for predictive biomarkers were recently developed by the Evaluation of Genomic Applications in Practice and Prevention (EGAPP) Working Group convened by the Centers for Disease Control: (1) Analytical validity; (2) Clinical Validity; and (3) Clinical Utility. We herein apply these paradigms to MGMT as a predictive biomarker.

#### Analytical validity

Pre-analytical and analytical factors that contribute to accuracy, reliability and reproducibility of the specific assay test for MGMT promoter methylation testing has become a controversial issue. There is still no consensus on the optimal method to be applied. Assessment of MGMT promoter methylation is difficult due to the complex nature of the techniques involved. To detect methylation, bisulfite treatment of the DNA is required, a process that may result in degradation of DNA and subsequent low success rates in PCR. This is further compounded by the fact that the most commonly available tissue for assessment is FFPE, and the DNA subsequently extracted is typically fragmented, again making PCR more difficult. Promoter methylation analysis by qualitative methyl-specific polymerase chain reaction (MSP) or semi-quantitative methyl-specific polymerase chain reaction (SQ-MSP), especially from FPPE tissue is technically demanding. MSP is the more limited because the methylation status of only a few CpG sites (i.e., those interfering with the PCR primer binding) can be interrogated at once. The technique also has the drawback of providing only a qualitative indication of the methylation status of the sites. Karayan-Tapon et al. (2010) evaluated MGMT promoter methylation using MSP, SQ-MSP, and pyrosequencing. The best predictive value for overall survival was obtained by pyrosequencing which is a technique that generates a quantitative measure of methylation and automatically calculates and reports percent methylation for each CpG site in the studied sequence, thus allowing detection of partially methylated CpG sites. The problem with pyrosequencing, however, is the different % cut-offs for methylation and clinical relevance of the cut-off chosen. Two regions tightly associated with patient survival were recently identified by Bady et al. (2012) using an Infinium methylation BeadChip. The latter of the two regions is within the 59 bp enhancer region, between the exon 1 and intron boundary of the *MGMT* gene. The majority of PCR assays, including the MSP and pyrosequencing, target this region.

#### Clinical validity

O6-methylguanine-DNA methyltransferase promoter methylation status is clinically well founded. In 2005, in a companion study conducted on tumor specimens from patients enrolled in the EORTC_26981/NCIC_CE.3 (Stupp et al., 2005b), Hegi et al. (2005) demonstrated a pronounced favorable response in patients whose tumors had lost MGMT activity via promoter methylation. The median survival for patients with methylated *MGMT* was 21.7 months compared to 12.7 months for patients with unmethylated *MGMT*. MGMT status can reliably distinguish two or more subgroups within a population that have different biological and clinical outcomes and has been used to stratify patient populations in a number of Phase II trials (Grossman et al., 2009; Fabi et al., 2010; Peereboom et al., 2010; Weiler et al., 2010; Lai et al., 2011). The strong prognostic role for *MGMT* promoter methylation in GBM treated with concurrent RT and TMZ was prospectively confirmed by the Radiation Therapy Oncology Group (RTOG) study 0525 (Ahluwalia, 2011).

The testing of patients for *MGMT* promoter methylation has been incorporated in numerous trials. A better response to cilengitide treatment in a phase I/IIa trial was observed in MGMT methylated patients (Stupp et al., 2010). In response, recruitment to the Phase III trial; Cilengitide, Temozolomide, and RT in Treating Patients With Newly Diagnosed GBM and Methylated Gene Promoter Status [CENTRIC]; NCT00689221 was restricted to patients with MGMT promoter methylation. The outcome of this trial is not yet known. The RTOG 0825 trial (mentioned earlier), examining the effect of bevacizumab administered with radiotherapy compared to conventional concurrent chemoradiotherapy (TMZ) in primary GBM also incorporated *MGMT* promoter methylation testing for all patients (Colman et al., 2010).

Two studies exploring the different treatment regimes in the elderly (patients aged over 60 years and diagnosed with a GBM): the German Neuro-oncology Group trial, NOA-08 (Wick et al., 2012) and the Nordic study (Malmstrom et al., 2012), also showed that *MGMT* promoter methylation was both prognostic and predictive for response to alkylating therapy. In both studies, survival was significantly improved in the *MGMT* methylated patients when treated with temozolomide, thus establishing *MGMT* as a predictive biomarker in this patient population. In contrast, *MGMT* promoter methylation appears to have no predictive capacity in anaplastic glioma. The NOA-04 trial showed a striking difference in PFS between patients with versus without *MGMT* promoter methylation who were treated with radiotherapy alone (Wick et al., 2012).

#### Clinical Utility

Clinical validity does not imply that a tumor biomarker assay should be used to care for patients. Prospective testing of elderly GBM patients for MGMT to determine if they should receive temozolomide is a significant advancement in clinical treatment. If *MGMT* methylated, up front temozolomide will be offered to this patient group. Importantly, these patients will be spared the radiotherapy which may lead to significant deficits including tiredness and memory loss. *MGMT* promoter methylation can also help to distinguish true tumor progression from pseudoprogression. Park et al. (2011) combined MS-MLPA and MSP methodologies for detecting *MGMT* methylation of which resulted in a diagnostic accuracy of 93% for the identification of pseudoprogression as opposed to radiological progression in GBM patients. It is critical to distinguish pseudoprogression because misinterpretation can lead to early treatment cessation or surgery.

The usefulness of testing for *MGMT* promoter methylation in all GBM patients in a routine clinical setting will come to fruition when an alternative treatment strategy is available. Until then, it is difficult and highly contentious for clinicians to base their decisions on whether or not they should withhold temozolomide treatment in patients with unmethylated MGMT and a good performance status.

With this recognition that an unmethylated MGMT promoter is associated with a poorer response to temozolomide, strategies have evolved to circumvent the resistance that MGMT confers. Numerous studies, including the multicenter RTOG 0525 have examined altered TMZ dosing regimes, but no significant survival benefits have been conferred (Brock et al., 1998; Wick et al., 2007, 2009; Perry et al., 2008; Ahluwalia, 2011). The MGMT unmethylated population of patients needs to be put on center stage for new treatment strategies.

## The Future of Biomarker Discovery and Implementation into the Clinic

We need to review where we are going wrong with biomarker discovery and development and identify the steps required to improve their clinical value and utility. First and foremost, we need to be asking the right questions. Is this biomarker involved in driving the cancer growth? Is this biomarker druggable? And of upmost importance, is it clinically relevant? Will this biomarker lead to a clinician altering their clinical decision? The collection of quality preserved tumor tissue that has undergone independent histological review will improve biomarker discovery. In the cases where biomarkers have been identified, standardized testing needs to be implemented.

### Importance of high quality tumor banking

The majority of biomarkers stem from studies on tumor tissue. Less than perfect tissue will lead to false-positives. Sub-standard tissue and data collection provided a significant obstacle to the TCGA effort. Standard operating procedures (SOPs) are not consistent between sites, and sometimes differ within single institutions. Methodologies for preserving tissue vary and times between tumor removal and time of processing fluctuate. Significant changes can occur between the time interval between tissue removal and processing. Despite this knowledge, in many centers, biorepositories remain underfunded. The collection of tissue needs to be taken seriously and investments are essential to promote basic, translational, and clinical research as well as social gain in terms of improved cancer care and economic development.

The same standards should apply to FFPE. However, unlike for frozen tissue, the time intervals taken for preservation are usually not recorded. Enrollment onto clinical trials is now often dependent upon receipt of FFPE tissue for central pathology and additional molecular tests such as Ion Torrent PMG sequencing which is a tailored platform to cater for FFPE tissue. Experience at one site in Australia revealed that FFPE specimens from patients operated between Monday-Thursday typically passed quality control and DNA extracted from these tissues tended to be suitable for sequencing. However, there were a sizeable number of FFPE samples that did not make the grade. These samples were usually from patients who were operated on a Thursday evening or a Friday. An investigation of the problem revealed that the specimens were kept in formalin solution for up to 72 h because samples were not processed over the weekend.

### Histological review

Histological classification of gliomas shows a significant inter-observer variability, particular in the distinction of astrocytomas vs. oligodendrogliomas and oligoastrocytomas (Kim et al., 2010). Similarly, the diagnosis of anaplastic astrocytoma can be very challenging (van den Bent, 2010). GBMs can easily be classified as anaplastic astrocytoma if the histological hallmarks necrosis and/or microvascular proliferation are not detected due to a sampling error. There is a need that all glioma diagnoses be complemented by a minimum set of genetic characteristic, including *IDH1* and *TP53* mutation, 1p/19q co-deletion, and *EGFR* amplification.

### Standardization of methodology for biomarker testing

There is also an apparent lack of standardization in the methods used for biomarker measurement. Assays for biomarkers need to be reliable. The assay needs to give identical results if repeated in the same or in another laboratory. The result needs to be the same, even when different methodologies are used. And finally, we need to ask whether the test provides added value to clinical practice. TCGA analyses have identified a high diversity of genes mutated within GBM. As prices drop with Next Generation sequencing, capabilities to better define genetic aberrations associated with response to a specific treatment will improve. Copy number aberrations (amplifications and deletions) and structural aberrations (intra-chromosomal rearrangements-inversions, inverted/tandem duplications) are not detected using traditional Sanger sequencing. Our ability to assess these aberrations must improve at the rate that targeted therapies emerge.

## Conclusion

To advance personalized medicine, a co-operative effort between cancer researchers and clinicians is needed. There is also insufficient collaboration within various scientific teams working on targeted therapies such as the TKIs and anti-angiogenics. Specific consideration needs to be paid to increasing sample sizes, sequencing entire genes, implementing robust methodologies and taking a holistic approach to understanding pathways. Cancer is multifaceted and it is our task to unravel these complexities. With highly integrated teams of multidisciplinary investigators, a better overall survival of GBM patients is achievable.

## Conflict of Interest Statement

The authors declare that the research was conducted in the absence of any commercial or financial relationships that could be construed as a potential conflict of interest.
